# Development and validation of a nomogram incorporating YpT stage for predicting ypN0 using multicenter data in clinically node-positive breast cancer

**DOI:** 10.1038/s41598-025-32167-8

**Published:** 2025-12-10

**Authors:** Hideo Shigematsu, Shinsuke Sasada, Junji Hashizume, Tatsunari Sasada, Shinji Ozaki, Midori Noma, Tomoyuki Yoshiyama, Goda Noriko, Ayumi Kawamata, Akiko Emi, Yuki Kaneko, Keiko Kajitani, Tomoko Itagaki, Morihito Okada

**Affiliations:** 1 Department of Surgical Oncology, Research Institute for Radiation Biology and Medicine, Hiroshima University, 1-2-3-Kasumi, Minami-ku, Hiroshima City, 734-8551 Japan; 2 Department of Breast Surgery, JA Onomichi General Hospital, Hiroshima, 722-8508 Japan; 3 Department of Breast and Endocrine Surgery, National Hospital Organization Higashihiroshima Medical Center, Hiroshima, 739-0041 Japan; 4 Department of Gastroenterological, Breast and Transplant Surgery, Hiroshima Prefectural Hospital, Hiroshima, 734-8530 Japan; 5 Department of Breast Surgery, National Hospital Organization Kure Medical Center and Chugoku Cancer Center, Hiroshima, 737-0023 Japan; 6 Department of Breast Surgery, Hiroshima City North Medical Center Asa Citizens Hospital, Hiroshima, 731-0293 Japan; 7 Department of Breast Surgery, JA Hiroshima General Hospital, Hiroshima, 738-8503 Japan

**Keywords:** Breast cancer, Neoadjuvant chemotherapy, Nomogram, YpN0, YpT stage, Cancer, Breast cancer, Predictive markers

## Abstract

Recent studies have demonstrated that breast pathological complete response (pCR) can be accurately diagnosed preoperatively using image-guided needle biopsy after neoadjuvant chemotherapy (NAC), underscoring the value of incorporating ypT stage into prediction models for axillary response (ypN0). Based on this evidence, we developed and externally validated a nomogram to predict ypN0 in patients with clinically node-positive (cN+) breast cancer treated with NAC. This retrospective, multicenter study included 609 patients with cN + breast cancer who underwent NAC followed by surgery. A nomogram was developed using a training cohort (n = 330) and validated in an independent cohort (n = 279). Diagnostic performance was evaluated using the area under the receiver operating characteristic curve (AUC), calibration plots, decision curve analysis (DCA), and positive predictive value (PPV). The nomogram incorporated five predictors: ypT stage, cN stage, hormone receptor status, HER2 status, and post-NAC cN stage. It showed strong discriminative ability, with bias-corrected AUCs of 0.858 in the training cohort and 0.855 in the validation cohort. Calibration and DCA confirmed good model performance. PPV exceeded 0.90 when the nomogram-predicted probability was ≥ 0.7 in both cohorts. This nomogram demonstrated high diagnostic accuracy for predicting ypN0 and may support the selection of candidates for axillary surgery de-escalation based on post-NAC biopsy findings. Prospective validation using biopsy-confirmed pCR is warranted.

**Supplementary Information:**

The online version contains supplementary material available at 10.1038/s41598-025-32167-8.

## Introduction

Neoadjuvant chemotherapy (NAC) has emerged as a cornerstone in the management of clinically node-positive (cN+) breast cancer as it not only increases the rate of breast conservation but also enables residual disease‐guided systemic therapy, which has been linked to improved prognostic outcomes^1–5^. Despite these advances in systemic therapy for breast cancer, a consensus on the optimal post‐NAC axillary management remains elusive in such patients^6–9^. Institutional variability in the indications for and extent of axillary surgery reflects ongoing challenges in the accurate assessment of residual nodal disease following NAC, a critical factor given the risks of lymphedema and upper‐limb dysfunction that are associated with axillary procedures.

Preoperative evaluation of nodal status in patients with cN + breast cancer after NAC relies predominantly on imaging modalities; however, a meta-analysis by Samiei et al. demonstrated that imaging-based assessments for pathological node negativity (ypN0) are limited by suboptimal diagnostic accuracy^10^. Although several predictive models that incorporate clinicopathological parameters—such as the tumor stage, biological subtype, and clinical response—have been developed, they have been primarily evaluated for their predictive performance rather than their diagnostic precision^11–13^. Given that safe omission of axillary surgery depends on reliable identification of ypN0, further validation of these models is imperative^14,15^.

Recent clinical efforts have focused on predicting breast pathological complete response (breast pCR) using needle biopsy of the tumor bed after NAC, as part of investigations into omission of surgery for patients who respond well to NAC^16–19^. Several studies have reported that image-guided needle biopsy can accurately diagnose breast pCR with high sensitivity and specificity. As this approach gains evidence, post-NAC needle biopsy may become a standard tool for determining eligibility for omission of surgery in the near future. This evolving ability to assess ypT stage preoperatively also strengthens its role as a clinically accessible predictor of axillary response.

In parallel, cohort studies involving patients with cN + breast cancer have shown that breast pCR correlates strongly with pathological node negativity, especially in hormone receptor (HR)-negative and HER2-positive subtypes^20,21^. Studies also reported a stronger association between ypT0 and ypN0 compared to ypTis^22,23^. These findings highlight the clinical relevance of ypT stage in predicting nodal response and support the development of a predictive model that incorporates ypT stage and other clinicopathological factors to improve patient selection for axillary surgery omission.

Given this evolving landscape, a predictive model incorporating ypT stage alongside other clinicopathological factors may have significant clinical utility in identifying cN + patients who could safely undergo less invasive axillary surgery. In this study, we developed and externally validated a nomogram incorporating ypT stage to predict ypN0 in cN + breast cancer patients treated with NAC.

## Materials and methods

### Study population

This observational multicenter study was conducted with ethical approval obtained from the institutional review boards of all participating institutions. We included consecutive cases of patients with cN + breast cancer who underwent NAC followed by definitive surgery from January 2006 to September 2024. cN + was defined as the histopathologically confirmed involvement of axillary lymph nodes based on fine-needle aspiration or core-needle biopsy. Patients with distant metastases, those who did not receive an anthracycline and/or taxane-based regimen, and cases with indeterminate HR and HER2 status were excluded. The primary objective of this study was to identify factors predictive of conversion to ypN0 following NAC and to construct a nomogram for estimation of the ypN0 probability. The training cohort consisted of patients from Hiroshima University Hospital, and external validation was performed with data from patients obtained from JA Onomichi General Hospital, Higashihiroshima Medical Center, Hiroshima Prefectural Hospital, Kure Medical Center, JA Hiroshima General Hospital, and Asa Citizens Hospital. Because this study entailed retrospective analysis of hospital database records, the requirement for written informed consent was waived; study details were publicly disclosed, and an opt‐out option was provided to patients. All methods were performed in accordance with the relevant guidelines and regulations.

### Data collection and analysis

Patient data were extracted from the hospital databases and electronic medical records of each participating institution. Collected clinical variables included age and preoperative T and N classifications, with staging determined according to the 7th edition of the American Joint Committee on Cancer staging system^24^. Pathological data from biopsy specimens were recorded, including the tumor histological type, grade, and the expression status of hormone receptors (estrogen receptor [ER] and progesterone receptor [PR]) and HER2. ER and PR statuses were evaluated via immunohistochemistry (IHC), and cases were deemed HR positive if ≥ 1% of tumor cells exhibited staining^25^. HER2 status was considered positive if IHC resulted in a score of 3 + or if in-situ hybridization demonstrated a gene amplification ratio greater than 2^26^. Post-NAC clinical nodal status (ycN) was primarily assessed by axillary ultrasound, with the disappearance of lymph node enlargement or normalization of nodal architecture (restoration of the fatty hilum and cortical thinning) interpreted as ycN0^27,28^. MRI or PET-CT findings were used as supplementary modalities according to each institution’s policy to support the ultrasound-based evaluation. Final ypT and ypN statuses were determined from surgical pathology reports. According to ypT stage, patients were classified as residual invasive carcinoma (non-ypT0/ypTis), noninvasive carcinoma (ypTis), or complete disappearance of the tumor (ypT0), while their nodal status was categorized as ypN0 or ypN + based on the presence or absence of residual-lymph-node metastases^29^.

### Neoadjuvant chemotherapy

NAC consisted of sequential anthracycline- and taxane-based regimens administered according to institutional protocols. Anthracycline regimens included AC or EC, and taxane regimens consisted of weekly paclitaxel or 3-weekly docetaxel, given sequentially in either order. Dose-dense schedules were also permitted, including ddAC/EC followed by weekly or q2-weekly paclitaxel. For HER2-positive disease, trastuzumab was co-administered with the taxane component, and pertuzumab was added following its approval for early-stage breast cancer. For triple-negative breast cancer, the KEYNOTE-522 regimen—comprising paclitaxel plus carboplatin with pembrolizumab, followed by pembrolizumab with anthracycline-based chemotherapy (AC or EC)—was used for stage II or higher cases after regulatory approval. Detailed regimen definitions, dosing, and schedules are provided in Supplementary Table S1.

### Endpoints and statistical analysis

Baseline characteristics of patients in the training and validation cohorts were compared using the Wilcoxon rank-sum test for continuous variables and the chi-square test for categorical variables. In the training cohort, logistic regression analysis was performed with ypN0 as the dependent variable. Variables that were statistically significant, as well as those that were not statistically significant but deemed clinically relevant, were subsequently incorporated into the nomogram^30^. The diagnostic performance of the nomogram was evaluated based on its discriminative ability, calibration, decision curve analysis (DCA), and diagnostic accuracy at various cutoff thresholds^31^. Discrimination ability was assessed by constructing receiver operating characteristic (ROC) curves and calculating the area under the curve (AUC) with 95% confidence intervals (CIs). Internal validation was performed using bootstrap resampling (*n* = 1000) to obtain bias-corrected AUC estimates. Calibration was evaluated using calibration curves and the Hosmer–Lemeshow goodness-of-fit test. Diagnostic accuracy at various thresholds was also determined, and the nomogram was further validated using an external cohort. All statistical analyses were performed using R version 4.3.2 (http://www.r-project.org; R Foundation for Statistical Computing, Vienna, Austria), with a two-sided significance level set at 5%.

## Results

### Characteristics of the training and validation cohorts

In this study, we used the data of 330 patients from Hiroshima University Hospital as the training cohort and those of 279 patients from other institutions as the validation cohort. Table 1 summarizes the baseline characteristics of the two cohorts. No significant differences were observed between the training and validation groups in terms of age (*p* = 0.74), histology (*p* = 0.23), clinical tumor size (*p* = 0.20), hormone receptor status (*p* = 0.13), ycN status (*p* = 0.93), pathological T status (*p* = 0.28), or pathological N status (*p* = 0.31). However, clinical nodal status (*p* = 0.032), histological grade (*p* < 0.001), and HER2 status (*p* < 0.001) differed significantly between the two groups. Specifically, the training cohort contained a higher proportion of patients with grade 3 tumors, whereas the validation cohort contained a higher proportion of patients with HER2-positive tumors.

**Table 1:** Characteristics of the training and validation cohorts.

	Training cohort (*n* = 330)	Validation cohort (*n* = 279)	*p* value
Age, Median (IQR)	56 (45.8–64.0)	56 (47.0–63.0)	0.74
Age Group			0.74
< 55 years	160 (48.5%)	139 (49.8%)	
≥ 55 years	170 (51.5%)	140 (50.2%)	
Race/Ethnicity	Asian (100%)	Asian (100%)	N/A
Histology			0.23
IDC, NOS	315 (95.5%)	260 (93.2%)	
Special	15 (4.5%)	19 (6.8%)	
cT Stage			0.20
T1	50 (15.2%)	30 (10.8%)	
T2	177 (53.6%)	140 (50.2%)	
T3	45 (13.6%)	42 (15.1%)	
T4	58 (17.6%)	67 (24.0%)	
cN Stage			0.032
N1	227 (68.8%)	199 (71.3%)	
N2	51 (15.5%)	37 (13.3%)	
N3	52 (15.8%)	43 (15.4%)	
Grade			< 0.001
Grade1	23 (7.0%)	60 (21.5%)	
Grade2	99 (30.0%)	81 (29.0%)	
Grade3	208 (63.0%)	138 (49.5%)	
Hormone Receptor Status			0.13
Positive	231 (70.0%)	179 (64.2%)	
Negative	99 (30.0%)	100 (35.8%)	
HER2 status			< 0.001
Positive	96 (29.1%)	127 (45.5%)	
Negative	234 (70.9%)	152 (54.5%)	
ycN Status			0.93
ycN0	207 (62.7%)	176 (63.1%)	
ycN+	123 (37.3%)	123 (36.9%)	
ypT Stage			0.28
ypT0	63 (19.1%)	68 (24.4%)	
ypTis	36 (10.9%)	27 (9.7%)	
non-ypT0/ypTis	231 (70.0%)	184 (66.0%)	
ypN status			0.31
ypN0	172 (52.1%)	157 (56.3%)	
ypN+	158 (47.9%)	122 (43.7%)	

*IQR* Interquartile range, IDC Invasive ductal carcinoma, *NOS* Not otherwise specified, *cT* Clinical tumor stage, *cN* Clinical nodal stage, *HER2* Human epidermal growth factor receptor 2, *ycN* Clinical nodal classification after neoadjuvant chemotherapy, *ypT* Pathological tumor classification after neoadjuvant chemotherapy, *ypN* Pathological nodal classification after neoadjuvant chemotherapy.

### Logistic regression analysis and nomogram construction for prediction of ypN0 in the training cohort

**Fig. 1:**
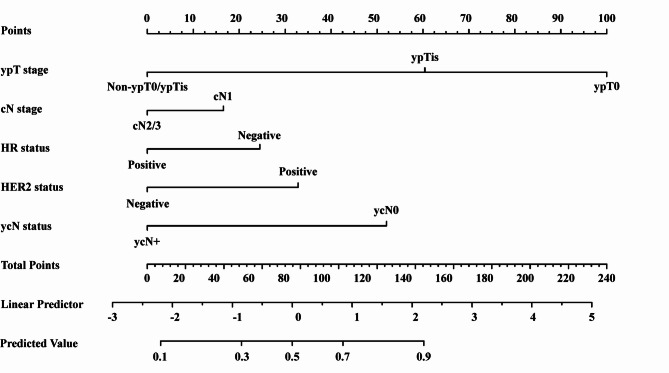
Nomogram for prediction of ypN0. The nomogram was developed using a training cohort of 330 patients and incorporates five variables: ypT stage, cN status, HR status, HER2 status, and ycN status. Each variable is assigned a corresponding score, which is summed to obtain the total score. This total is mapped to a linear predictor and the predicted probability of ypN0. As an example, for a patient with ypT0, cN1, HR-negative, HER2-positive, and ycN0 status, the total score is approximately 210 points, corresponding to a predicted probability of ypN0 of ≈ 0.92.

Based on the logistic regression results in the training cohort (Table 2) and on clinical relevance, we selected five factors—ypT stage, clinical nodal status, HR status, HER2 status, and ycN status—to construct a nomogram (Fig. 1). In this nomogram, each predictor is allocated a score on the uppermost axis in line with the value on its own axis. By summing these points across all five variables, a total score is obtained on the “Total Score” axis; this score can be translated into a predicted probability of ypN0 by using the corresponding scale at the bottom of the nomogram. In clinical practice, a physician would identify a patient’s scores for each of the five factors, mark the associated score for each category, sum them to determine the patient’s overall score, and draw a line to determine the estimated probability of having ypN0.

**Table 2:** Logistic regression analysis for prediction of ypN0 in the training cohort.

Factor	Univariate OR (95% CI)	*P* value	Multivariate OR (95% CI)	*P* value
Age				
≤ 55 years	1	-	1	-
> 55 years	0.80 (0.52–1.23)	0.31	0.87 (0.49–1.56)	0.64
Histological Type				
IDC, NOS	1	-	1	-
Special type	1.05 (0.37–3.07)	0.92	2.17 (0.59–7.90)	0.24
cT Stage				
cT1, 2	1		1	-
cT3, 4	1.46 (0.92–2.34)	0.11	1.19 (0.63–2.26)	0.58
cN Stage				
cN1	1		1	
cN2, 3	1.64 (1.02–2.63)	0.039	1.87 (0.92–3.79)	0.080
Hormone Receptor Status				
Positive	1	-	1	-
Negative	2.86 (1.75–4.77)	< 0.001	2.20 (1.13–4.30)	0.021
HER2				
Negative	1	-	1	-
Positive	5.93 (3.43–10.7)	< 0.001	2.72 (1.34–5.51)	0.006
Grade				
Grade1, 2	1	-	1	-
Grade3	1.57 (1.00–2.46.00.46)	0.05	1.39 (0.76–2.53)	0.29
ycN Status				
ycN+	1	-	1	-
ycN0	6.77 (4.13–11.4)	< 0.001	8.42 (2.92–10.1)	< 0.001
ypT Stage				
ypT0	1	-	1	
ypTis	11.5 (4.67–34.6)	< 0.001	7.78 (2.57–23.5)	< 0.001
non-ypT0/ypTis	37.0 (13.2–155.0)	< 0.001	25.7 (7.26–90.8)	< 0.001

*OR* Odds ratio, *CI* Confidence interval, *IDC* Invasive ductal carcinoma, not otherwise specified, *cT* Clinical tumor stage, *cN* Clinical nodal stage, *HER2* Human epidermal growth factor receptor 2, *ycN* Clinical nodal classification after neoadjuvant chemotherapy, *ypT* Pathological tumor classification after neoadjuvant chemotherapy, *ypN* Pathological nodal classification after neoadjuvant chemotherapy.

**Table 3:** Diagnostic performance of the nomogram for prediction of ypN0 at various risk thresholds.

Threshold	TN	FP	FN	TP	Sensitivity	Specificity	PPV	NPV
A. Training cohort (n = 330)								
0.9	155	3	100	72	0.42	0.98	0.96	0.61
0.8	151	7	84	88	0.51	0.96	0.93	0.64
0.7	149	9	80	92	0.53	0.94	0.91	0.65
0.6	136	22	58	114	0.66	0.86	0.84	0.70
B. Validation cohort (n = 279)								
0.9	116	6	78	79	0.50	0.95	0.93	0.60
0.8	115	7	68	89	0.57	0.94	0.93	0.63
0.7	115	7	65	92	0.59	0.94	0.93	0.64
0.6	106	16	43	114	0.73	0.87	0.88	0.71

*TN* True negative, *FP* False positive, *FN* False negative, *TP* True positive, *Sens* Sensitivity, *Spec* Specificity, *PPV* Positive predictive value, *NPV* Negative predictive value.

### Internal validation of the nomogram

**Fig. 2:**
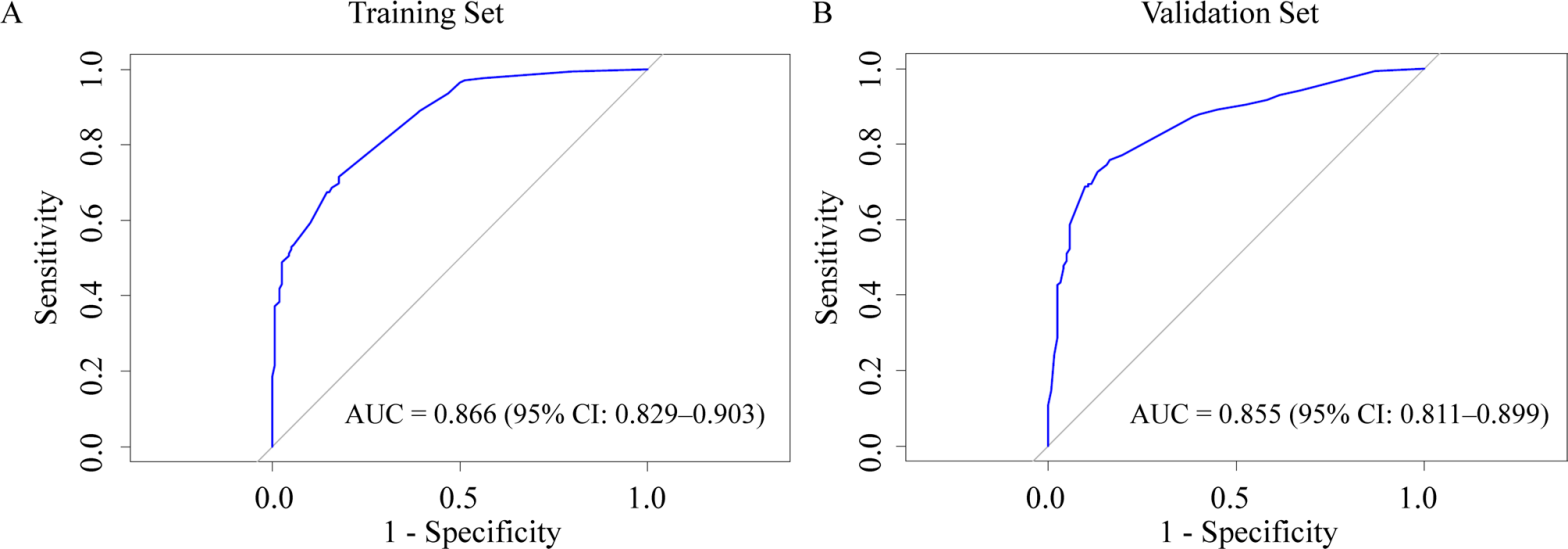
ROC curves of the nomogram for prediction of ypN0. The ROC curves demonstrate the nomogram’s discriminative performance, with the x-axis representing the false positive rate and the y-axis representing the true positive rate.

**Fig. 3:**
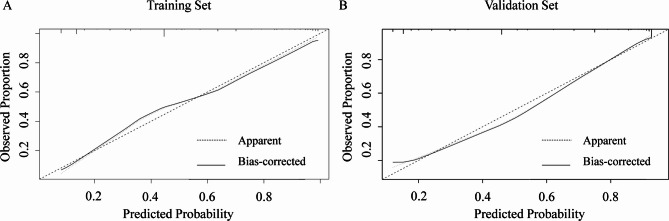
Calibration plots of the nomogram for prediction of ypN0. (**A**) Calibration plot for the training cohort. The apparent and bias-corrected calibration curves (bootstrap resampling) are shown. The dashed line indicates perfect calibration. The Hosmer–Lemeshow test revealed no significant lack of fit (χ^2^ = 8.19, df = 7, *p* = 0.32). (**B**) Calibration plot for the validation cohort, showing similar results (χ^2^ = 12.07, df = 8, *p* = 0.15).

**Fig. 4:**
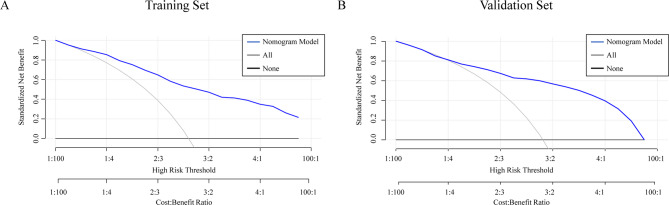
DCA of the nomogram for prediction of ypN0. The gray and black curves correspond to the “treat-all” and “treat-none” strategies, respectively.

ROC curve analysis for the diagnostic value of the nomogram yielded an AUC of 0.866 (95% CI: 0.829–0.903), with a bootstrap-corrected AUC of 0.858 (Fig. 2A). The calibration curve demonstrated close agreement between observed and predicted values (Fig. 3A), and the Hosmer–Lemeshow test revealed no significant lack of fit (χ^2^ = 8.19, degrees of freedom [df] = 7, *p* = 0.32). DCA visualized the model’s net benefit across various risk thresholds (Fig. 4A). Table 3A summarizes the diagnostic performance of the nomogram at various risk thresholds for the prediction of ypN0 in the training cohort. At thresholds of 0.9, 0.8, 0.7, and 0.6, the sensitivity was 0.42, 0.51, 0.54, and 0.66, respectively, while the specificity was 0.98, 0.96, 0.94, and 0.86, respectively. The positive predictive value (PPV) exceeded 0.90 at thresholds of 0.9 (0.96), 0.8 (0.93), and 0.7 (0.91).

### External validation of the nomogram

External validation of the nomogram was performed using a validation cohort. The results of the ROC curve analysis, calibration curve, and DCA are presented in Figs. 2B and 3B, and 4B. The AUC was 0.855 (95% CI: 0.811–0.899). The calibration curve demonstrated good agreement between predicted and observed probabilities, and the Hosmer–Lemeshow test revealed no significant lack of fit (χ^2^ = 12.07, df = 8, *p* = 0.15). DCA was used to visualize the model’s net benefit across various risk thresholds. In a post hoc analysis, the diagnostic performance of the nomogram was evaluated across different probability thresholds to assess its predictive accuracy for ypN0. Table 3B summarizes the diagnostic performance of the nomogram at various risk thresholds for the prediction of ypN0 in the validation cohort. At thresholds of 0.9, 0.8, 0.7, and 0.6, the sensitivity was 0.50, 0.57, 0.59, and 0.73, respectively, while the specificity was 0.95, 0.94, 0.94, and 0.87, respectively. The positive predictive value (PPV) remained consistently high, exceeding 0.90 at thresholds of 0.9 (0.93), 0.8 (0.93), and 0.7 (0.93).

## Discussion

In this study, we developed and validated a nomogram for the prediction of ypN0 in patients with cN + breast cancer who were treated with NAC. Unlike previous models, our nomogram uniquely incorporates the presence or absence of a pCR alongside other clinicopathological parameters. The model had high diagnostic performance, with AUC values of 0.866 in the training cohort and 0.855 in the external validation cohort. Notably, the nomogram exhibited a particularly high PPV across multiple risk thresholds, suggesting its potential clinical utility in the selection of candidates for de-escalation of axillary surgery.

Previous ypN0-prediction models for cN + breast cancer have yielded moderate to good discriminative performance; however, further improvements are necessary to ensure the safe omission of axillary surgery. The model developed and externally validated by Vila et al. achieved an AUC of 0.78–0.82, but it relied solely on clinicopathological factors and did not incorporate dynamic parameters—such as the tumor reduction rate or clinical response—that reflect the effectiveness of NAC^11^. Although Corsi et al. developed a model that incorporates a clinical complete response as a predictor of ypN0, it had only moderate diagnostic accuracy (sensitivity: 71%, specificity: 73%, AUC = 0.77)^12^. In contrast, the model developed by Huang et al. had a high discriminative performance (AUC = 0.85), using a nomogram that integrated post-NAC ultrasound features of axillary lymph nodes with clinicopathological features^13^. However, a breast ultrasound-based determination of a complete response has a high false-positive rate, and the use of multiple ultrasound parameters for axillary lymph nodes poses challenges in terms of reproducibility and generalizability. In light of these findings, future efforts should be focused on the incorporation of objective measures that accurately reflect NAC effectiveness into prediction models to enhance diagnostic accuracy and ensure the safe omission of axillary surgery.

Unlike previous nomogram models that primarily relied on pre-NAC clinicopathological parameters, our model uniquely incorporates the post-treatment ypT stage—an emerging, biopsy-assessable indicator—thereby bridging the transition toward biopsy-based surgical de-escalation strategies. Recent retrospective analyses have consistently shown that breast pCR, particularly ypT0, is strongly associated with ypN0, especially in HR-negative and HER2-positive subtype^20–23^. These findings support the clinical relevance of ypT stage as a surrogate marker for nodal response after NAC. As breast pCR is defined by pathological assessment of the surgical specimen, it has conventionally been available only postoperatively. However, recent studies focusing on omission of surgery for NAC responders have demonstrated that it can be reliably assessed using image-guided needle biopsy of the tumor bed^16–19^. A representative study by Tasoulis et al. demonstrated the feasibility of using image-guided vacuum-assisted biopsy to preoperatively assess breast pCR after NAC^16^. In a multicenter pooled analysis involving 166 patients, they showed that applying a standardized protocol yielded a false-negative rate of 3.2%, a negative predictive value of 97.4%, and an overall diagnostic accuracy of 89.5%. These findings support the reliability of image-guided biopsy in identifying patients who achieve ypT0 and may inform patient selection for de-escalated surgical approaches.

To achieve a high PPV for predicting ypN0, we developed a nomogram that incorporates ypT stage in patients with cN + breast cancer. The model demonstrated strong discriminative ability and good calibration, and in a post-hoc analysis evaluating diagnostic performance across probability thresholds, both the training and validation cohorts showed that a predicted probability of ≥ 0.7 corresponded to a PPV ≥ 0.90, indicating a false-negative rate below 10%. This level of diagnostic performance is clinically meaningful, as a PPV ≥ 0.90 corresponds to a false-negative rate (FNR) below 10%—a widely accepted benchmark for safely omitting axillary surgery. Landmark axillary de-escalation trials such as ACOSOG Z1071, SENTINA, and SN FNAC were similarly designed to maintain the FNR below this threshold^6,7,32^. Importantly, all variables included in our nomogram—such as HR status, HER2 status, and clinical nodal response—are routinely available in standard clinical practice, reinforcing its practical applicability. HR-negative and HER2-positive tumors typically exhibit higher proliferative activity and greater chemosensitivity than HR-positive/HER2-negative tumors. The strong association of these subtypes with ypN0 likely reflects their intrinsic molecular responsiveness to cytotoxic and HER2-targeted therapy. Although recent AI-based prediction models using large datasets have demonstrated excellent discriminative performance, our clinically interpretable nomogram provides a transparent, easily applicable tool for bedside decision-making. Future integration of deep learning or machine-learning frameworks using larger multicenter datasets may further enhance model generalizability.

Despite the promising performance of our model, its primary limitation lies in the use of ypT stage derived from surgical specimens. Because ypT0 currently requires pathological confirmation after surgery, the model’s applicability is confined to postoperative prediction and cannot yet be directly applied to preoperative decision-making. However, as image-guided or vacuum-assisted biopsy techniques for confirming breast pCR continue to evolve, similar nomograms based on biopsy-confirmed pCR may enable preoperative prediction of ypN0 in the near future. In addition, the retrospective and multicenter design, spanning a long enrolment period (2006–2024), may have introduced heterogeneity in imaging methods, systemic therapy regimens, and pathological assessment across institutions and time periods. Because of the limited sample size within each era, adjustment or stratification by treatment period was not feasible, which may have affected the statistical robustness and generalizability of the findings. Although our nomogram showed consistent performance across cohorts, further prospective validation using biopsy-based pCR and long-term clinical outcomes will be essential to establish its generalizability and clinical utility in guiding axillary surgery de-escalation.

In conclusion, our nomogram incorporating ypT stage provides a robust and clinically practical tool for predicting ypN0 in patients with cN + breast cancer treated with NAC. With consistently high PPVs exceeding 0.90, the model meets the diagnostic performance threshold traditionally required for safely omitting axillary surgery. As the preoperative assessment of breast pCR via image-guided biopsy becomes increasingly feasible, this model may serve as a foundation for individualized axillary management strategies in the era of surgical de-escalation. Future prospective studies are warranted to validate its performance in biopsy-based settings and to support its implementation in clinical decision-making.

## Supplementary Information

Below is the link to the electronic supplementary material.

Supplementary Material 1

## Data Availability

The datasets used during the current study are available from the corresponding author upon reasonable request. Declarations
