# Supplementary material for: Development and validation of a nomogram incorporating YpT stage for predicting ypN0 using multicenter data in clinically node-positive breast cancer
Source: Sci Rep. 2025 Dec 10;16:2344. doi: 10.1038/s41598-025-32167-8 (PMC12816637; doi:10.1038/s41598-025-32167-8)
Supplement: Supplementary file 1 — Supplementary Material 1 [file 41598_2025_32167_MOESM1_ESM.docx]

Supplementary Table S1. Neoadjuvant Chemotherapy Regimens Used in the Study

Detailed definitions, typical dosing, and schedules of neoadjuvant chemotherapy regimens administered across participating institutions.

| Subtype | Regimen (acronym) | Components & dosing (typical) | Schedule |
| --- | --- | --- | --- |
| All | AC | Doxorubicin 60 mg/m² + Cyclophosphamide 600 mg/m² | q3w × 4 |
| All | EC | Epirubicin 90 mg/m² + Cyclophosphamide 600 mg/m² | q3w × 4 |
| All | ddAC | Doxorubicin 60 mg/m² + Cyclophosphamide 600 mg/m² with G‑CSF support | q2w × 4 |
| All | ddEC | Epirubicin 90 mg/m² + Cyclophosphamide 600 mg/m² with G‑CSF support | q2w × 4 |
| All | PTX (weekly) | Paclitaxel 80 mg/m² | Weekly × 12 |
| All | DTX | Docetaxel 75 mg/m² | q3w × 4 |
| All | ddPTX | Paclitaxel 175 mg/m² | q2w × 4 |
| HER2-positive | TH | Taxane (PTX weekly ×12 or DTX 75 mg/m² q3w ×4) + Trastuzumab | Per taxane schedule |
| HER2-positive | THP | Taxane (as above) + Trastuzumab + Pertuzumab (added after approval for early-stage breast cancer) | Per taxane schedule |
| HR-negative/HER2-negative (Stage II+) | KEYNOTE‑522 | Paclitaxel 80 mg/m² weekly + Carboplatin (AUC 1.5 weekly or AUC 5 q3w) + Pembrolizumab, followed by AC or EC + Pembrolizumab | Per protocol |

Abbreviations: AC, doxorubicin + cyclophosphamide; EC, epirubicin + cyclophosphamide; PTX, paclitaxel; DTX, docetaxel; dd, dose-dense; G-CSF, granulocyte colony-stimulating factor; TH, taxane + trastuzumab; THP, taxane + trastuzumab + pertuzumab; HR, hormone receptor; HER2, human epidermal growth factor receptor 2, neoadjuvant chemotherapy.
